# Predictors of adenoma size and location in primary hyperparathyroidism

**DOI:** 10.1007/s00423-021-02179-9

**Published:** 2021-04-30

**Authors:** Barbara Filser, Verena Uslar, Dirk Weyhe, Navid Tabriz

**Affiliations:** Medical Campus University of Oldenburg, School of Medicine and Health Sciences, University Hospital for Visceral Surgery, Pius-Hospital Oldenburg, Georgstr. 12, 26121 Oldenburg, Germany

**Keywords:** Hyperparathyroidism, Hypercalcemia, Parathyroidectomy, Intraoperative parathyroid hormone

## Abstract

**Purpose:**

In primary hyperparathyroidism (PHPT), intraoperative localization of the parathyroid adenoma can be challenging, especially in cases of negative preoperative imaging. Since a focused unilateral parathyroidectomy has benefits compared to a conventional bilateral neck exploration, the question arises whether adenoma size prediction can facilitate a targeted approach. We investigated whether single parathyroid adenoma size can be estimated using preoperative parathyroid hormone (PTH), calcium, and phosphate in patients with PHPT. Preoperative imaging accuracy was evaluated.

**Methods:**

The data of 156 patients who underwent curative parathyroidectomy for single adenoma PHPT were analyzed retrospectively. Information obtained included laboratory data, imaging results, intraoperative data, and final pathology. Imaging accuracy was analyzed descriptively. The association between preoperative biochemical markers and adenoma dimensions was investigated using Spearman’s correlation coefficient and multivariable regression modeling.

**Results:**

Cervical ultrasound correctly predicted adenoma laterality in 95.5%, sestamibi scintigraphy in 80.6%, both had lower true-positive rates for quadrant prediction. Patients with negative imaging results showed higher thyroid volumes than those with positive results. Adenoma volume was positively correlated with preoperative PTH (*p* < 0.001) and calcium (*p* < 0.001) and negatively correlated with preoperative phosphate (*p* = 0.001). Using these preoperative biochemical markers and patient age and BMI, adenoma volume can be significantly predicted using the multivariable regression algorithm.

**Conclusion:**

Cervical ultrasound is superior to scintigraphy for predicting adenoma location and should be the first-choice imaging method, but both methods may be limited by increased thyroid volume. Large adenomas are more likely with higher PTH, higher calcium, and lower phosphate levels. In cases of undetermined adenoma location, an estimation of adenoma volume via our algorithm could corroborate sonographic volume measurements of the suspected adenoma.

## Introduction

Primary hyperparathyroidism (PHPT) describes an autonomous overproduction of parathyroid hormone (PTH) in one or more parathyroid glands. Clinical symptoms arise due to the resulting chronic hypercalcemia; the most frequent manifestations being nephrolithiasis and decreased bone mineral density. However, the majority of patients nowadays are diagnosed due to an incidental finding of elevated calcium on routine laboratory tests and do not show symptoms. Diagnosis is confirmed by the laboratory profile of elevated PTH, elevated calcium, and often decreased phosphate [1–4].

Surgical adenoma excision is the only definitive treatment and is always recommended in symptomatic patients. It is furthermore recommended in asymptomatic patients who are younger than 50 years, have significant hypercalcemia or show signs of osteoporosis or renal complications on imaging investigations [1, 3, 5–7]. Since the most common cause for PHPT is a single adenoma, a focused unilateral approach may be used to minimize operative time and risks [8, 9]. However, this approach requires both sensitive preoperative localization methods and reliable means of intraoperative adenoma verification.

For operative planning, cervical ultrasound and ^99m^Tc-sestamibi scintigraphy are most commonly used. However, both methods are limited in multiglandular hyperplasia and multiple adenomas, and have further specific limitations [8–14]: sonographic imaging is highly dependent on sonographer experience. Small adenomas are difficult to identify, as are retropharyngeal or retroesophageal glands and ectopic glands in the mediastinum. Visualization may be impeded in patients with concurrent thyroid disease and in obese patients [5, 8, 12, 15]. Scintigraphy is less prone to limitations due to operator dependence than sonography. However, this technique is less useful for quadrant prediction than for laterality prediction [8, 16]. False positive results may arise from benign and malign thyroid nodules, thyroid inflammation, and cervical lymphadenopathy, and false negatives may occur with adenomas weighing less than 0.6–0.8 g [8, 11].

Computed tomography (CT) and magnetic resonance imaging (MRI) are less frequently used, typically in cases in which prior imaging is inconclusive or contradictory or an ectopic adenoma is suspected [5, 8]. It is important to note that negative imaging does not preclude a patient from surgery [7]; however, an adenoma of undetermined location requires an operative exploration of all parathyroid glands, which could lead to increased morbidity [8, 9]. Therefore, the question arises whether preoperative laboratory markers can help determine adenoma size for a focused pre- or intraoperative exploration. Several previous studies have investigated correlations between preoperative biochemical markers and adenoma dimensions with diverging findings [17–24]. A significant correlation of preoperative PTH and parathyroid gland size has been shown repeatedly [17–21, 23, 24]. Of those studies, all but one showed a concomitant correlation of serum calcium and tumor dimensions [18]. Phosphate repeatedly showed not to be linked to adenoma size [17, 19, 22]. As a result of the heterogeneity of the available studies with regard to design and sample size and the diverging findings, the role of biochemical markers as predictors of parathyroid dimensions prior to surgery remains indeterminate.

Intraoperative PTH (IOPTH) assays are a widely used method for intraoperative adenoma verification with excellent overall operative success rates of 97–99%. The most common criteria require a 50% drop in hormone 10 or 20 min after adenoma excision to confirm operative success [25, 26].

We evaluated the accuracy of the imaging techniques performed in our patients by means of a descriptive analysis of 156 successful operations with definitive confirmation of adenoma location. Furthermore, we aimed to clarify the role of biochemical markers as predictors of adenoma size by determining associations between preoperative PTH, calcium, and phosphate levels on the one hand, and parathyroid volume on the other using correlation analyses and multiple linear regression.

## Methods

### Patients

**Fig. 1:**
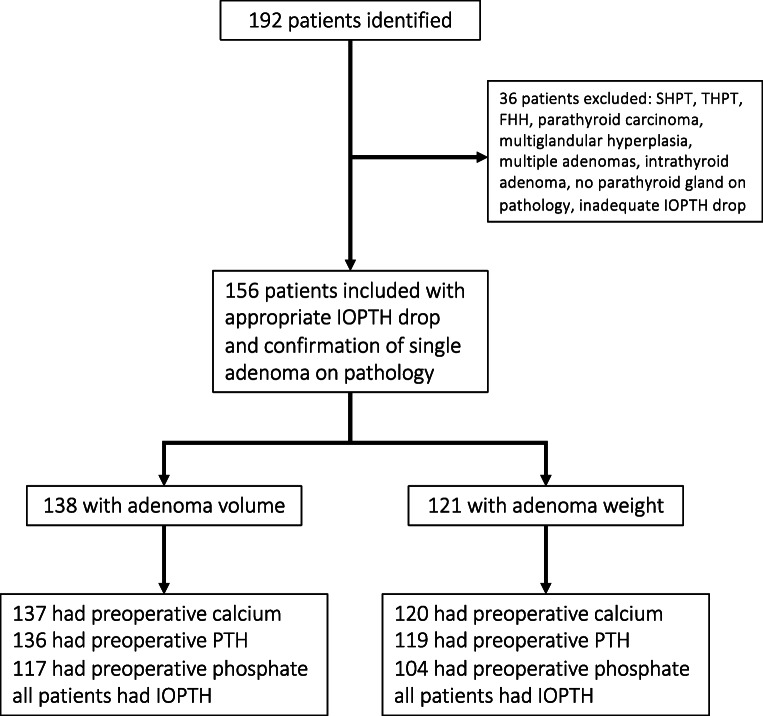
Flowchart of patients identified and included in the analysis. We excluded 36 patients who did not meet the inclusion criteria of successful parathyroid surgery for a single adenoma. Patients missing the named laboratory values were excluded from the respective correlation analyses. SHPT, secondary hyperparathyroidism; THPT, tertiary hyperparathyroidism; FHH, familial hypocalciuric hypercalcemia; IOPTH, intraoperative parathyroid hormone

We conducted a monocentric retrospective study of patients undergoing parathyroidectomy between January 2016 and December 2019 at our institution, a certified center for thyroid and parathyroid surgery. To identify the patient collective, we consulted a prospectively maintained database. Additional laboratory or surgical variables not included in the original database were retrospectively acquired and added. We included all patients with single adenoma PHPT, in which operative success was confirmed by adequate IOPTH dynamics and the pathology report (Fig. 1). By this approach, we hoped to identify factors which could be helpful in cases when surgery is expected to be difficult (for example in unknown adenoma location). Exclusion criteria were secondary or tertiary hyperparathyroidism, familial hypocalciuric hypercalcemia, multiglandular hyperplasia or multiple adenomas, intrathyroid adenomas, and unsuccessful surgery (i.e., no parathyroid gland on pathology, inadequate IOPTH drop).

Patient characteristics, surgical data (type and duration of procedure), duration of stay, preoperative, intraoperative and postoperative laboratory values, preoperative imaging results, intraoperative findings, final pathology, and complications (with respective management) were obtained. Adenoma weight (g) and dimensions (mm or cm) were retrieved from the pathology reports. Adenoma volume was calculated using the formula for an ellipsoid object ($V=\frac{4}{3}\ \pi abc$ with *a*, *b*, *c* constituting the semi-axes).

### Preoperative imaging

Preoperative imaging results were assembled from both the in-house reports and the reports of external experts in endocrinology, radiology, and nuclear medicine. According to the available records, patients receiving scintigraphy imaging had dual phase scintigraphy, with both early imaging 10–20 min after tracer administration and a late phase image after 90–180 min, in some cases, complementary SPECT was performed. All patients received cervical sonography performed by one of our surgeons prior to operative planning.

### Surgery

Patients with indicative imaging received a focused unilateral exploration. In cases of inconclusive or discordant imaging, a bilateral exploration was performed. IOPTH was used in all patients to confirm excision success intraoperatively. Operative success was defined as a > 50% drop 20 min after gland excision, consistent with the Rome protocol [25]. Inappropriate PTH decay dynamics lead to further exploration and excision of macroscopically suspect gland tissue with subsequent IOPTH measurements. All PTH and IOPTH measurements were undertaken using the Elecsys® PTH STAT Assay.

### Statistical analysis

Descriptive analysis of patient characteristics and the variables of interest were performed. Preoperative imaging results were analyzed descriptively and compared to intraoperative findings. Accuracy was indicated as true-positive rates for laterality prediction and quadrant prediction. Correlation analyses were performed using nonparametric Spearman’s rank correlation coefficient between parathyroid volume (ml) as dependent variable and preoperative PTH (pg/ml), calcium (mmol/l), and phosphate (mmol/l) as instrumental variables. Scatter plots were created to illustrate the relationship between the variables. The levels of significance for the correlation analyses were controlled for false discovery using the Benjamini and Hochberg method [27]. The association between preoperative parameters and adenoma volume was investigated using multiple linear regression modeling. The model offering the highest R-square value was selected. Before interpreting the results, assumptions for linear regression were checked using Durbin-Watson test, collinearity statistics as well as scatter plots and normality plots. All analyses were conducted using SPSS 26 by IBM. Statistical significance was defined as *p* ≤ 0.05.

## Results

### Descriptive analysis

We report a female predominance of approximately 3 to 1 (73.7% (*n* = 115) female, 26.3% (*n* = 41) male), and a mean age of 59 years (Table 1). Five patients (3.2%) received a simultaneous partial or total thyroidectomy for thyroid comorbidity. Four patients (2.6%) received secondary exploration after IOPTH values indicated remaining hyperfunctioning parathyroid tissue. 41.7% (*n* = 65) of all single adenomas were located in the lower right quadrant, 34.6% (*n* = 54) in the lower left quadrant, 15.4% (*n* = 24) in the left upper quadrant, 8.3% (*n* = 13) in the right upper quadrant. With regard to complications, we recorded one case of cervical hematoma (0.6%) which was surgically decompressed, as well as three cases of unilateral recurrent laryngeal nerve injury (1.9%). Seventy-eight patients (50%) reported transient tingling paresthesia, though the laboratory workup showed mild hypocalcemia in merely 8 patients (5.1%), half of which had experienced sensory disturbances. Treatment consisted of oral calcium substitution. Persistence of hypocalcemia or sensory disturbances was reported in none of the patients included in this study.

**Table 1:** Patient characteristics, endocrine profile, and adenoma characteristics

	**N**	**Median**	**Mean**	**SD**	**Min–max**
Age (years)	156	60.5	59.2	13.7	22–84
BMI (kg/m^2^)	156	27.1	28.1	5.8	18.7–48.9
Operation time (min)	156	28.0	34.3	20.6	7.0–133
Duration of stay (days)	156	5.0	5.0	1.0	3.0–11
Thyroid volume (ml)	136	14.0	16.3	11.5	0–72.0
TSH (ug/ml)	155	1.52	1.71	1.33	0.01–12.95
T3 (pg/ml)	151	3.08	3.06	0.53	1.30–5.98
T4 (ng/dl)	151	1.12	1.18	0.75	0.63–10.12
Preoperative PTH (pg/ml)	154	138.1	183.1	137.4	59.0–1046.8
Preoperative calcium (mmol/l)	155	2.81	2.85	0.21	2.44–3.56
Preoperative phosphate (mmol/l)	131	0.83	0.84	0.17	0.43–1.34
Pre-excision PTH (pg/l)	156	130.4	173.1	150.6	55.3–1510.0
Post-excision PTH (pg/ml)	156	24.6	31.2	28.0	5.8–238.4
% IOPTH drop	156	82.8	80.0	11.3	39.0–96.0
Adenoma weight (g)	121	0.850	1.571	2.076	0.180–13.000
Adenoma volume (ml/cm^3^)	138	0.940	2.022	3.073	0.084–19.635
Postoperative minimum calcium (mmol/l)	155	2.3	2.3	0.2	1.9–3.0

### Imaging accuracy

Out of our 156 patients, 155 received preoperative cervical ultrasound (the remaining ultrasound result could not be obtained due to an incomplete medical record) and 67 received preoperative sestamibi imaging. Cervical ultrasound correctly predicted adenoma laterality in 95.5% (*n* = 148), sestamibi scintigraphy did in 80.6% (*n* = 54). Quadrant prediction rates were lower (76.8% (*n* = 119) for sonography and 64.2% (*n* = 43) for scintigraphy). Five patients received additional MR imaging, with a 40% (*n* = 2) correct laterality prediction, and one patient received PET CT imaging, with correct laterality prediction.

**Fig. 2:**
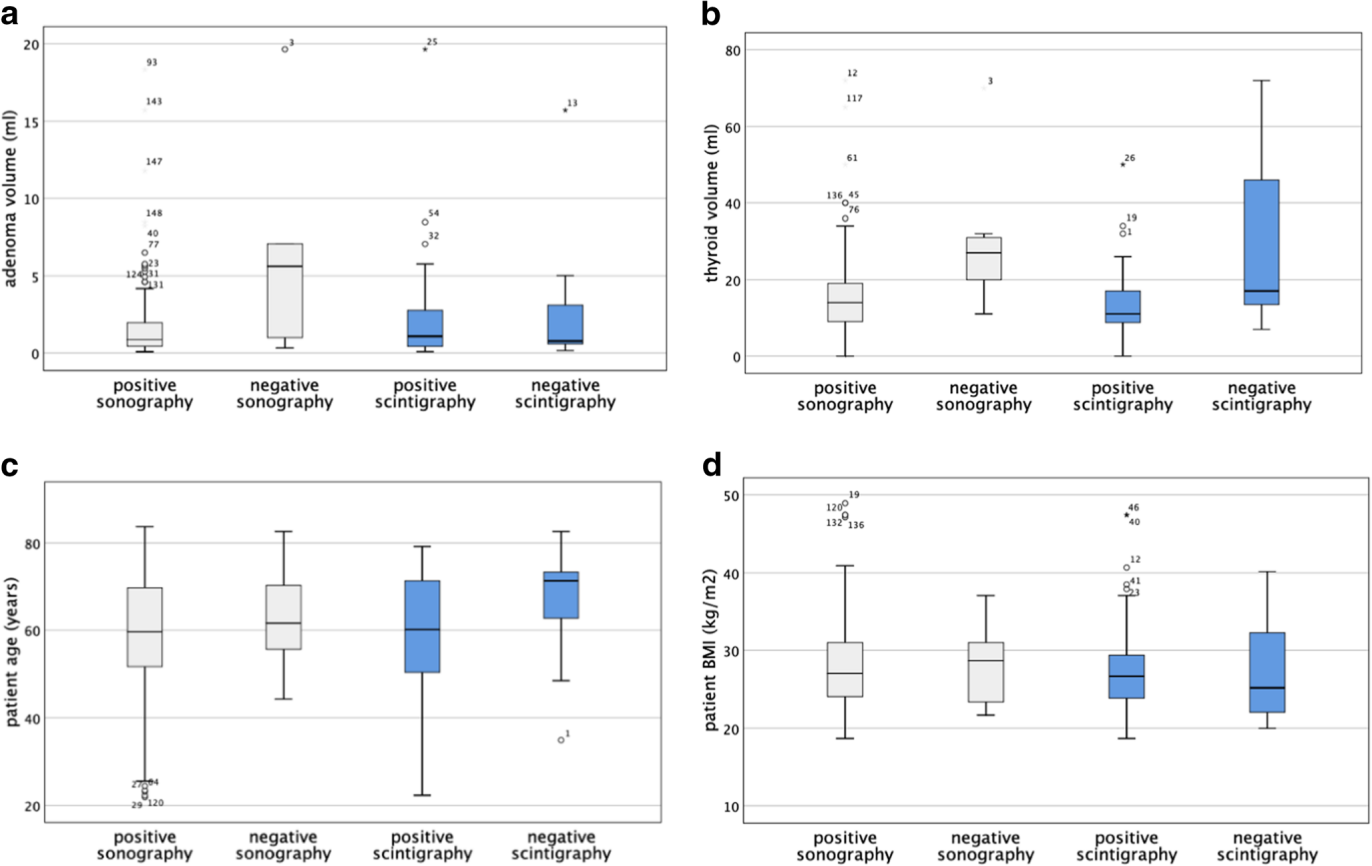
Boxplots comparing patient characteristics and adenoma characteristics of patients who had negative imaging results with patients who had positive imaging results (positive sonography: *n* = 148, negative sonography: *n* = 7, positive scintigraphy: *n* = 54, negative scintigraphy: *n* = 13). **a** Adenoma volume (ml). **b** Thyroid volume (ml). **c** Patient age (years). **d** BMI (kg/m^2^)

Of the seven adenomas missed on ultrasound, six received additional scintigraphy which correctly identified four adenomas (66.7%). Of the 13 adenomas missed on scintigraphy, all received additional sonography which correctly identified eleven adenomas (84.6%). With the exception of 2 patients, in which neither sonography nor scintigraphy identified the correct adenoma location and 1 patient who received no additional imaging besides sonography, 153 patients had at least 1 positive imaging result. For patients with positive and negative imaging results, we observe no consistent difference in adenoma volume, age, or BMI. However, patients with negative sonography and scintigraphy had higher mean, minimum, and maximum thyroid volumes (see Fig. 2).

### Predictors of adenoma size

Parathyroid volume based on our calculation and parathyroid weight indicated on the pathology report showed a very strong positive linear correlation (*ρ* = 0.906, *p* < 0.001).

**Fig. 3:**
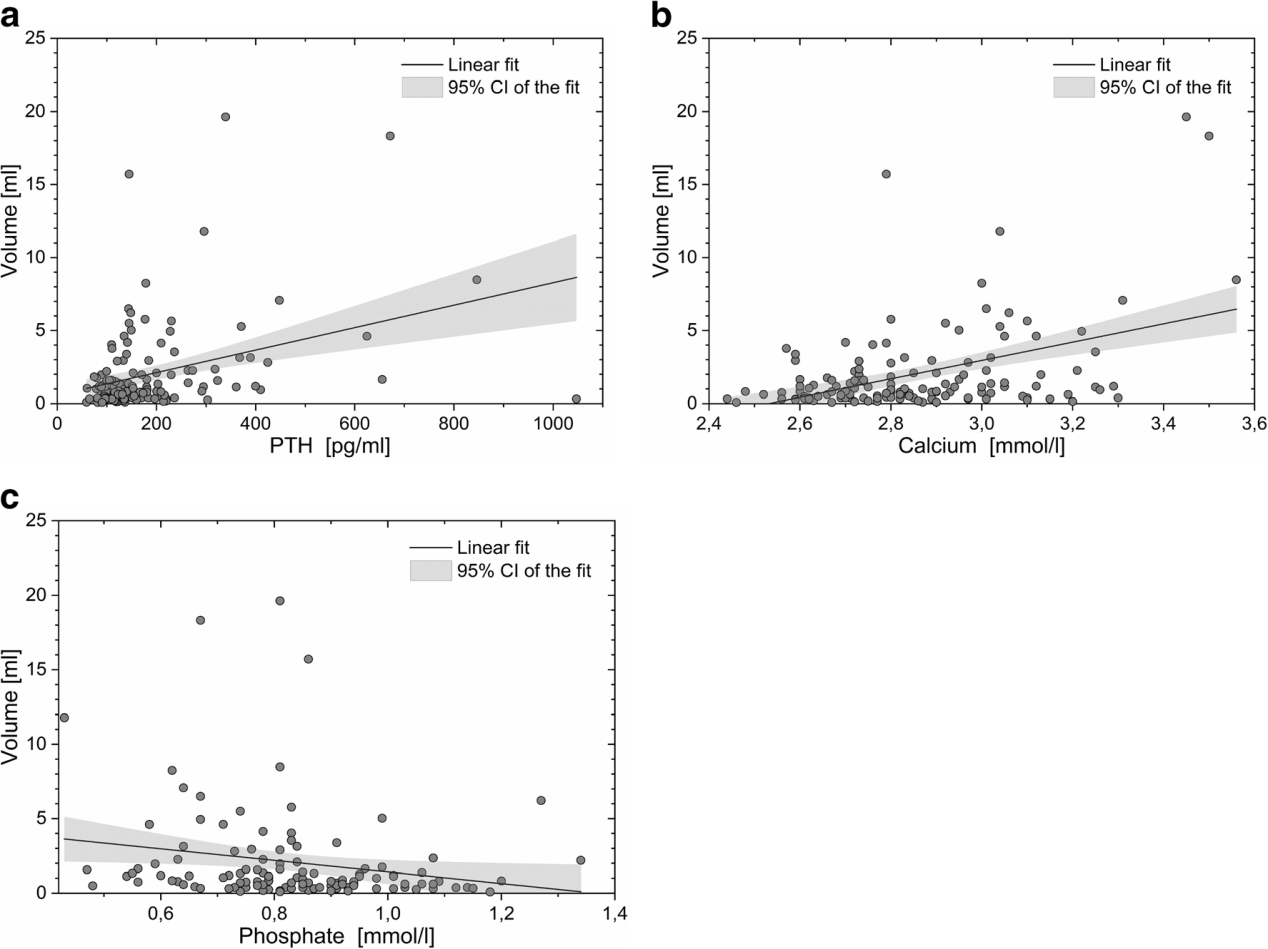
Scatterplots displaying the relationship between biochemical markers and parathyroid volume. Rho (*ρ*) indicates Spearman’s correlation coefficient. **a** PTH vs parathyroid volume. **b** Total calcium vs parathyroid volume. **c** Phosphate vs parathyroid volume

Of the predictors included in our analysis (Fig. 3), PTH had the strongest correlation with parathyroid volume (*ρ* = 0.342, *p* < 0.001). Calcium was positively correlated with parathyroid volume (*ρ* = 0.295, *p* < 0.001), and phosphate was negatively correlated with parathyroid volume (*ρ* = − 0.296, *p* = 0.001).

The associations established above were further investigated using multivariable linear regression modeling. The regression model with PTH, calcium, phosphate, and patient age and BMI significantly explained 26.0% of the variance in adenoma volume (*R*^2^ = 0.260, *F*(5, 111) = 7.804, *p* < 0.001). Variables significantly associated with adenoma volume were PTH (*B* = 0.004, *p* = 0.024) and calcium (*B* = 5.448, *p* < 0.001) (see also Table 2). The residuals showed no autocorrelation (Durbin-Watson *d* = 1.712), and there was no critical collinearity; however, the residuals showed heteroscedasticity. Based on the multivariable regression model, adenoma volume can be estimated using the following algorithm: volume = – 13.657 + 0.004*PTH + 5.448*calcium – 1.308*phosphate – 0.014*age + 0.041*BMI.

**Table 2:** Multiple linear regression model (*n* = 117) with adenoma volume as dependent variable

	***B***	**SE(B)**	**β**	**95% CI for** ***B***	**Significance**
**Constant**	− 13.657	4.420		− 22.415, − 4.900	0.003
**PTH**	0.004	0.002	0.206	0.001, 0.008	0.024
**Calcium**	5.448	1.312	0.370	2.849, 8.047	< 0.001
**Phosphate**	− 1.308	1.640	− 0.069	− 4.557, 1.942	0.427
**Patient age**	− 0.014	0.020	− 0.058	− 0.053, 0.025	0.485
**BMI**	0.041	0.049	0.071	− 0.056, 0.139	0.400

*B* unstandardized coefficient, *SE* standard error of estimate, *β* standardized coefficient, *CI* confidence interval

## Discussion

Imaging sensitivities of ultrasound and sestamibi scintigraphy as preoperative localization techniques have been subject to numerous previous investigations. In our study, we found a correct laterality prediction rate of 95.5% and a quadrant prediction rate of 76.8% for single adenomas using cervical ultrasound imaging. Sestamibi scintigraphy showed a true-positive rate of 80.6% for laterality prediction. Cheung et al. indicate a pooled sensitivity of 76.1% for ultrasound imaging and 78.9% for sestamibi scintigraphy in their meta-analysis [10]. The difference in the sensitivities of ultrasound imaging may be explained by the fact that we excluded multiple adenomas and multiglandular hyperplasia in our analysis, which are known to limit sensitivity [10, 14]. In previous studies, low-weight adenomas were more often missed on both imaging techniques [11, 28]. In our patient collective, parathyroid gland weight was not consistently lower in patients with negative imaging. However, we observed that those patients more often showed high thyroid volumes, which is consistent with previous studies suggesting that concomitant nodular thyroid disease and goiter are important limiting factors [15, 29–34].

Ultrasound is inexpensive, non-invasive, does not involve radiation exposure, and allows an evaluation of concomitant thyroid pathology, and if done by an experienced sonographer, is superior to sestamibi scintigraphy, as has been shown in this and several other studies [10, 14, 35–37]. For these reasons, Korwar et al. suggest to use ultrasound as sole primary imaging and reserve sestamibi scintigraphy and other modalities for cases of negative ultrasound or suspected ectopic adenomas [35]. Other authors have argued that concordant results of multiple imaging studies improve intraoperative localization rates [38]. As our results reveal high sonographic accuracy even in cases of negative scintigraphy, we support the approach as suggested by Korwar et al.

As preoperative imaging cannot offer an accurate localization in all cases, an indication of adenoma dimensions can provide useful information. We found a moderate correlation of PTH with adenoma volume, which has been shown in several other studies, with the exception of a study performed by Randhawa et al. [18–24]. Furthermore, our study shows a moderate positive correlation of preoperative calcium and adenoma dimensions, which aligns with all but two previous investigations [18–24]. Preoperative serum phosphate was seldomly included in correlative analyses. In our study, a weak negative correlation of phosphate and adenoma volume was found for the first time in a patient collective of 156 patients, contradicting 3 previous studies in which a significant correlation was refuted [17, 19, 22]. Since our analysis comprises a larger sample size, our finding of a correlation is plausible; however, its role as a predictor of adenoma size has yet to be clarified.

The established associations between PTH, calcium, phosphate, and adenoma dimensions were further investigated using multiple linear regression. We formulated an algorithm which can be used to estimate adenoma volume using preoperative biochemical markers and patient characteristics. Variables significantly associated with adenoma dimensions were PTH and calcium, whereas phosphate, patient age, and BMI showed no significant association. This method has been previously applied by Leong et al., who included calcium, logarithmic PTH, and patient age in their regression model as predictors of adenoma weight as useful preoperative information [20].

Since our regression algorithm predicts adenoma volume rather than weight, we propose a complementary role for preoperative sonography instead. For instance, if the predicted adenoma volume and the sonographic volume measurement of the suspected adenoma align, a correct localization could be assumed. Naturally, as our algorithm is based on a retrospective analysis of patients treated at our center in the past years, this method is merely a suggestion that has not yet been validated prospectively. Another limitation of our method is the statistical limitation of our regression model due to heteroscedasticity, which describes a heterogenous variance of the residuals. The consequence of heteroscedasticity is potential bias in the calculation of the standard error, thereby making tests of significance imprecise [39].

We acknowledge the limitations of the presented data due to the retrospective study design. However, as a certified center for thyroid and parathyroid surgery, patient data is prospectively acquired, offering us a relatively comprehensive dataset. Based on this, we hope to offer further insight into common problems in the management of primary hyperparathyroidism and discuss an approach which may facilitate a focused approach to parathyroidectomy.

## Conclusion

In single adenoma primary hyperparathyroidism, a minimally invasive surgical approach is the preferable choice to minimize operative time and risks. However, intraoperative adenoma identification may be difficult, especially if preoperative imaging results give no clear indication of adenoma location. When opting for a unilateral approach, additional information may be taken into consideration: large adenomas are more likely in patients with higher PTH, higher calcium, and lower phosphate levels. Most adenomas in our study were located in the lower right quadrant, followed by the lower left quadrant. Cervical sonography proved superior to sestamibi imaging for prediction of adenoma location; however, both methods may be limited by increased thyroid volume. We propose an algorithm that gives an approximation of expected adenoma volume using preoperative biochemical markers and patient age and BMI. Our algorithm aims to optimize preoperative ultrasound imaging, since the calculated volume via our algorithm and the sonographically measured volume of a suspected structure could be compared. Our hope is that as preoperative localization techniques improve, the need for operative exploration of all four parathyroid glands and the increased risks thereof will decrease. We would therefore like to start a discussion as to whether size estimation can increase the diagnostic value of preoperative ultrasound.
